# The association of healthy eating index with periodontitis in NHANES 2013–2014

**DOI:** 10.3389/fnut.2022.968073

**Published:** 2022-08-09

**Authors:** Xin-yu Li, Ming-zhe Wen, Yu-hua Xu, Yu-chen Shen, Xi-tao Yang

**Affiliations:** ^1^Department of Interventional Therapy, Multidisciplinary Team of Vascular Anomalies, Shanghai Ninth People's Hospital, Shanghai Jiao Tong University, Shanghai, China; ^2^Department of Neurosurgery, Shanghai Ninth People's Hospital, Shanghai Jiao Tong University, Shanghai, China

**Keywords:** periodontal disease, healthy eating index, periodontitis, dietary structure, HEI

## Abstract

**Background:**

Periodontal disease is very common worldwide and is one of the main causes of tooth loss in adults. Periodontal disease is characterized by chronic inflammation that can destroy adjacent alveolar bone and lead to a loss of periodontal ligaments. Although previous studies have found that a daily diet can influence the development of periodontal disease (e.g., a diet low in carbohydrates and rich in vitamins C and D and fiber can have a protective effect). Periodontal disease may present as gingivitis or periodontitis. However, studies on the role of healthy eating index in periodontitis are lacking. The purpose of this study was to assess the association between healthy eating index and periodontitis.

**Methods:**

We analyzed data collected from participants in the National Health and Nutrition Examination Survey (NHANES), a nationally representative survey conducted in 2-year cycles from 2013 to 2014. As part of our analysis, we developed multivariate logistic regression models to examine the independent association between the healthy eating index and periodontitis. We evaluated the significance of association using odds ratios (OR) with 95% confidence intervals (95%CI).

**Results:**

Individuals with a lower total healthy eating index had a higher prevalence of periodontitis. Adjusted multivariate regression models showed that a higher healthy diet index was associated with a lower prevalence of periodontitis (OR = 0.69, 95% CI: 0.55–0.86, *P* < 0.05).

**Conclusion:**

The results of the study showed that dietary structure was associated with the prevalence of periodontitis. Patients with a higher healthy eating index had a lower prevalence of periodontitis. These findings will need to be confirmed by longitudinal, prospective studies in the future.

## Introduction

Periodontal disease is a complex inflammatory disease caused by pathogenic plaque biofilm that affects not only the gums and bone but also the alveolar bone, resulting in bleeding gums, periodontal pocket formation, and loss of attachment, which can lead to loosening and loss of teeth in the long run (1). According to statistics from the United States, 47% of adults older than 30 years have chronic periodontitis, 30% have moderate periodontal disease and 8.5% have severe periodontal disease (2, 3). Previous studies have suggested that several factors such as lifestyle, obesity and metabolic syndrome and genetic factors increase the risk of periodontitis (4). Healthy eating habits promote health, while unhealthy eating habits are associated with a variety of chronic diseases (5). Based on dietary guidelines for Americans (DGA) recommendations, Healthy Eating Index (HEI) indicates how closely a diet adheres to the DGA, with a higher score indicating better compliance (6, 7). The HEI-2015 consists of 13 dietary components. Nine adequate components: including total fruit, all fruit, all vegetables, greens and legumes, whole grains, dairy products, all protein foods, seafood and vegetable proteins, and fatty acids. Four moderate components: including refined grains, sodium, added sugars and saturated fats. Each element has a maximum score of 5 or 10 points. The total score is 100, and the higher the score means the closer to the recommended range or number (8). HEI is a valid and reliable measure of dietary quality for different population subgroups and interventions related to nutrition (8). However, there are limited studies on the role of HEI in periodontitis. The purpose of this study was to assess the prevalence of periodontitis between those participants with a lower HEI and those with a higher HEI.

## Methods

### Study population

Data were drawn from the 2013–2014 NHANES. All adults aged 30 or older who had a permanent tooth were eligible for full-mouth periodontal examinations (9). Participants in NHANES completed a questionnaire at home, followed by a physical examination and interviews at a mobile exam center (MEC). Data collected at the clinical examinations were standardized with minimal site-specific bias. Because only a subset of NHANES participants underwent MEC examinations, we included only those who reported a complete dental examination. Dietary quality was obtained from 24 h dietary recalls and was assessed by HEI (2015) scores. We also included other demographic variables (including age, gender, race, educational attainment, smoking status, and alcohol use status) and BMI (Body Mass Index). Ultimately, we included 3.001 participants for the next step of analysis.

### Study variables

#### Socio-demographic characteristics

Socio-demographic characteristics were set as age, gender (male/female), race (Mexican American; white; black and other), education level (less than high school; high school and college or above), smoking status (former smoker; never smoker and now smoker), diabetes (DM; no), drinking status (never; former; mild; moderate and heavy) and poverty income ratio (PIR).

The classification of smoking status is based on the following criteria (10): A never smoker is defined as an adult who has never smoked or smoked <100 cigarettes in their lifetime; Smokers who reported smoking ≥100 cigarettes in their lifetime and were currently non-smokers were identified as former smokers; whereas current smokers were defined as those who smoked ≥100 cigarettes on some days or every day in their lifetime. Never drinkers were defined as those who reported drinking <12 drinks; ever drinkers were defined as those who had more than 12 drinks in their lifetime but not in the past year; current drinkers were further classified as light, moderate and heavy current drinkers. Heavy current drinkers were defined as ≥3 drinks per day for women and ≥4 drinks per day for men, with five or more binge drinking days per month; moderate drinkers were defined as ≥2 drinks per day for women and ≥3 drinks per day for men, with ≥2 binge drinking days per month. Light drinkers: did not meet the above criteria (11, 12). In the US, PIR is defined as the ratio between a household's self-reported income and the poverty line. According to the PIR, we categorize family income into three levels: low (PIR <1.35), medium (1.35 ≤ PIR <3.0) and high (PIR ≥3.0) (13). BMI was divided into four categories (14): underweight (BMI <18.5), normal weight (BMI = 18.5–24.9), overweight (BMI = 25–29.9) and obese (BMI >30.0). An individual who reports themselves to have diabetes mellitus or who uses antidiabetic medication is considered to have diabetes.

#### Periodontitis

The full-mouth periodontal examination was performed by a calibrated dentist who assessed the periodontal status of the participants. Periodontal examinations comprised probing depths (PD) and clinical attachment levels (AL) at the MEC. Supplementary Table 1 shows the criteria for classification according to periodontal status (15).

#### HEI

Using an automated multiple-pass method, the NHANES dietary data include 24-h dietary recalls collected by computer-assisted dietary interview software. According to the United States Department of Agriculture (USDA) Food and Nutrient Database for Dietary Studies, the nutrient values for each food were assigned (16). Food components, with the exception of fatty acids, are scored on a density basis (per 1,000 kcal or as a percentage of energy). Fatty acids are expressed as a ratio of unsaturated to saturated fatty acids (17). Dietary components and standards for scoring are shown in Supplementary Table 2. For the adequacy components, better quality is reflected in higher scores. For the moderation components, a lower intake will result in a higher score.

### Statistical analysis

For descriptive analyses, continuous variables were described by means and standard deviations; categorical variables were described by frequencies and percentages. HEI-2015 classification according to quartiles (quartile 1: <25th percentile, quartile 2: ≥25 to 50th percentile, quartile 3: ≥50 to 75th percentile, quartile 4: ≥75th percentile) (18). We used Fisher's exact test to assess the difference in distributions of categorical variables (19). We then presented descriptive statistics comparing the prevalence of periodontitis by HEI. We examined the association between HEI and periodontitis using invariable and multivariable logistic regression model while adjusting for age, sex, race, smoking, drinking, education level and BMI.

## Results

### Population characteristics

**Figure 1:**
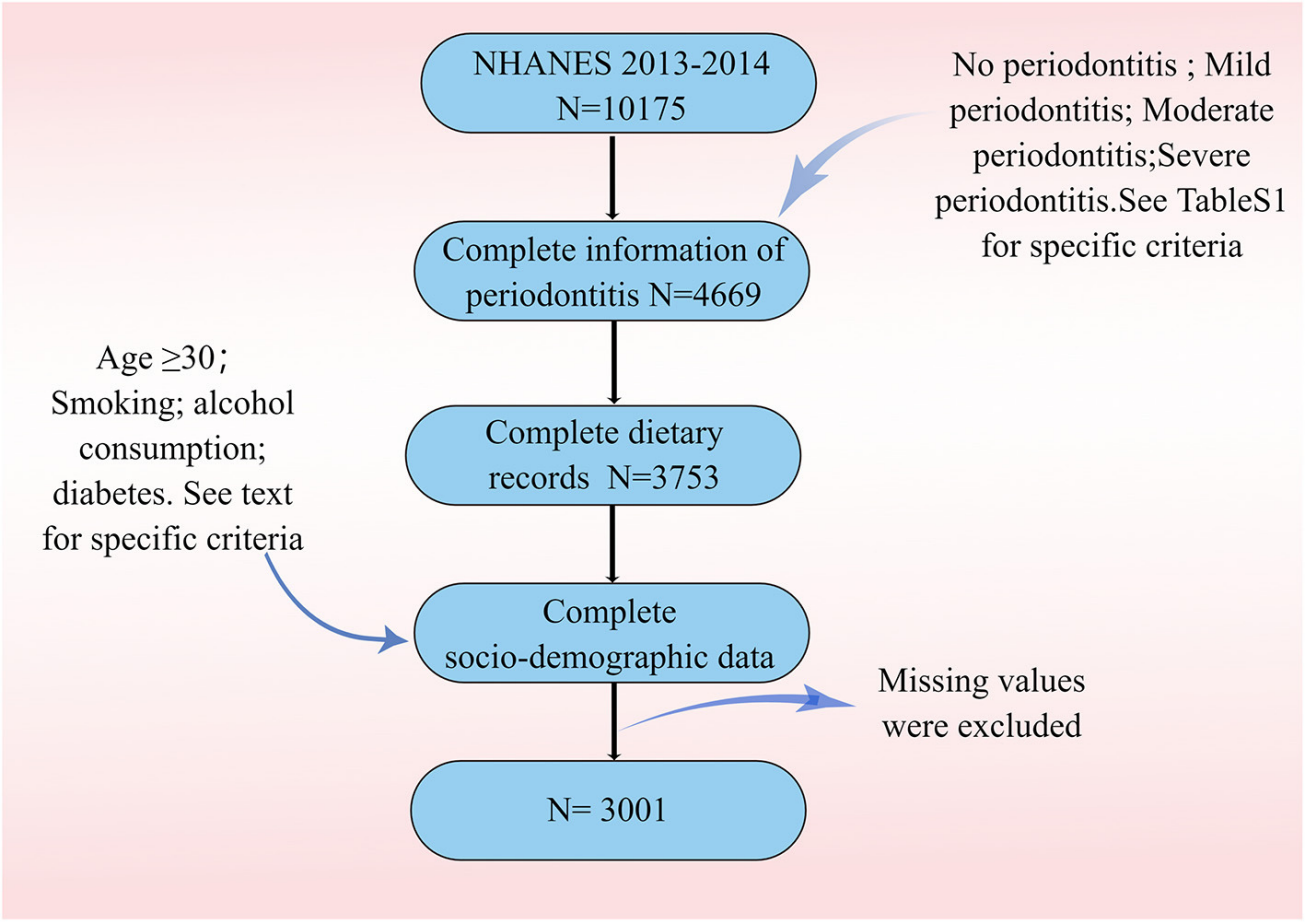
Flowchart of the study population. (ID:PORYR171ff).

Figure 1 describes study recruitment and inclusion/exclusion criteria. Multiple socio-demographic groups had statistically significant higher rates of periodontitis (Table 1). The average age of participants with periodontitis was 55 years, higher than those who did not have periodontitis (49 years). In the four HEI groups, the prevalence of severe periodontitis was 32.7, 25.9, 25.5, and 15.9% in that order. The prevalence of periodontitis is highest in non-Hispanic white, followed by non-Hispanic black. At the same time, the prevalence of periodontitis was significantly higher among participants with low PIR and among those with high school or less education. Periodontitis is most prevalent in light drinkers and least prevalent in never drinkers. Finally, the incidence of periodontitis is higher in those with a history of smoking compared to non-smokers. The prevalence of periodontitis was higher in participants with DM than in those without DM (58.6 vs. 40.2%). In terms of BMI, obese participants had the highest number of people with periodontitis, but the prevalence was not statistically different.

**Table 1:** Characteristics of the overall target population according to periodontitis.

**Variables**	**Total (*n* = 3,001)**	**No (*n* = 1,715)**	**Mild (*n* = 37)**	**Moderate (*n* = 998)**	**Severe (*n* = 251)**	***p***
**HEI**, ***n*** **(%)**						<0.001
Q1	750 (25.0)	402 (23.4)	12 (32.4)	254 (25.5)	82 (32.7)	
Q2	750 (25.0)	410 (23.9)	7 (18.9)	268 (26.9)	65 (25.9)	
Q3	750 (25.0)	422 (24.6)	12 (32.4)	252 (25.3)	64 (25.5)	
Q4	751 (25.0)	481 (28)	6 (16.2)	224 (22.4)	40 (15.9)	
Age, Mean ± SD	52.1 ± 14.3	49.4 ± 13.9	43.4 ± 12.6	55.9 ± 14.4	55.9 ± 11.9	<0.001
**Sex**, ***n*** **(%)**						<0.001
Female	1523 (50.7)	985 (57.4)	17 (45.9)	441 (44.2)	80 (31.9)	
Male	1478 (49.3)	730 (42.6)	20 (54.1)	557 (55.8)	171 (68.1)	
**DM**, ***n*** **(%)**						<0.001
DM	435 (14.6)	180 (10.6)	6 (16.2)	211 (21.2)	38 (15.1)	
No	2551 (85.4)	1525 (89.4)	31 (83.8)	782 (78.8)	213 (84.9)	
**Race**, ***n*** **(%)**						<0.001
Black	570 (19.0)	259 (15.1)	9 (24.3)	219 (21.9)	83 (33.1)	
Mexican American	382 (12.7)	168 (9.8)	5 (13.5)	168 (16.8)	41 (16.3)	
Other Race	667 (22.2)	401 (23.4)	11 (29.7)	212 (21.2)	43 (17.1)	
White	1382 (46.1)	887 (51.7)	12 (32.4)	399 (40)	84 (33.5)	
**PIR**, ***n*** **(%)**						<0.001
High	1071 (35.7)	782 (45.6)	10 (27)	237 (23.7)	42 (16.7)	
Low	883 (29.4)	378 (22)	10 (27)	386 (38.7)	109 (43.4)	
Medium	1047 (34.9)	555 (32.4)	17 (45.9)	375 (37.6)	100 (39.8)	
**Education**, ***n*** **(%)**						<0.001
College or above	1819 (60.6)	1230 (71.7)	21 (56.8)	482 (48.3)	86 (34.3)	
High school	655 (21.8)	297 (17.3)	11 (29.7)	267 (26.8)	80 (31.9)	
Less high school	527 (17.6)	188 (11)	5 (13.5)	249 (24.9)	85 (33.9)	
**Smoke**, ***n*** **(%)**						<0.001
Former	776 (25.9)	400 (23.3)	5 (13.5)	305 (30.6)	66 (26.3)	
Never	1686 (56.2)	1103 (64.3)	26 (70.3)	463 (46.4)	94 (37.5)	
Now	539 (18.0)	212 (12.4)	6 (16.2)	230 (23)	91 (36.3)	
**Alcohol**, ***n*** **(%)**						<0.001
Former	511 (17.0)	235 (13.7)	4 (10.8)	221 (22.1)	51 (20.3)	
Heavy	515 (17.2)	274 (16)	9 (24.3)	174 (17.4)	58 (23.1)	
Mild	1115 (37.2)	683 (39.8)	15 (40.5)	334 (33.5)	83 (33.1)	
Moderate	458 (15.3)	294 (17.1)	4 (10.8)	127 (12.7)	33 (13.1)	
Never	402 (13.4)	229 (13.4)	5 (13.5)	142 (14.2)	26 (10.4)	
**BMI**, ***n*** **(%)**						0.348
Normal	756 (25.2)	457 (26.6)	9 (24.3)	235 (23.5)	55 (21.9)	
Obese	1188 (39.6)	653 (38.1)	18 (48.6)	413 (41.4)	104 (41.4)	
Overweight	1028 (34.3)	592 (34.5)	10 (27)	338 (33.9)	88 (35.1)	
Underweight	29 (1.0)	13 (0.8)	0 (0)	12 (1.2)	4 (1.6)	

### Outcome and exposure factors

According to the total scores, participants were divided into four quartiles and periodontitis ever accounted for 27.1% in Q1, 26.4% in Q2, 25.5% in Q3, and 21% in Q4. Participants with HEI scores in the highest quartile had a lower prevalence of periodontitis than those in the lowest quartile (21.0 vs. 27.1%). At the same time, our results suggest a gradual decrease in the prevalence of periodontitis as the HEI rises. Also, in terms of the severity of periodontitis, participants in the top quartile of HEI scores had a lower proportion (15.9 %) of severe periodontitis than participants in the other groups. Supplementary Table 3 showed the distributions of HEI-2015 components by categories of HEI.

### Multivariate regression analysis

All four models showed a negative correlation between HEI and the prevalence of periodontitis in 2015, based on the quartiles of HEI (Table 2). That is, logistic regression analysis demonstrated that higher quartiles of 2015 HEI were associated with a lower prevalence of periodontitis. Compared to the lowest quartile of the HEI-2015 population, the fourth quartile had a lower prevalence of periodontitis in model 1 (OR = 0.53; 95%CI 0.43–0.66), model 2 (OR = 0.75; 95%CI 0.59–0.95) and model 3 (OR = 0.69; 95%CI 0.55–0.86). *P* for trend was <0.05 in all models. The results of the multivariate regression analysis of the 2015 HEI components showed that that scores of fatty acids, seafood and plant proteins, whole grains; total fruit and total vegetables were all significantly associated with periodontitis (Table 3). Table 4 shows the actual effect of smoking, age and gender, with the results suggesting that the absence of a smoking history has a protective effect against periodontitis.

**Table 2:** Association of HEI-2015 with periodontitis.

**Exposure**	**Unadjusted model**	**Model 1**	**Model 2**	**Model 3**
HEI_Q1	1 (Ref)	1 (Ref)	1 (Ref)	1 (Ref)
HEI_Q2	0.96 (0.78–1.17); 0.678	0.89 (0.72–1.1); 0.293	0.99 (0.79–1.24); 0.935	0.99 (0.8–1.23); 0.938
HEI_Q3	0.9 (0.73–1.1); 0.299	0.78 (0.63–0.96); 0.02	0.91 (0.72–1.14); 0.414	0.91 (0.73–1.14); 0.399
HEI_Q4	0.65 (0.53–0.8); <0.001	0.53 (0.43–0.66); <0.001	0.75 (0.59–0.95); 0.016	0.69 (0.55–0.86); 0.001
*P*-value for trend	<0.001	<0.001	0.045	0.036

*Model 1: adjusted for gender, age, race*.

*Model 2: Model 1+ adjusted for education, PIR*.

*Model 3: Model 2+smoke, DM, alcohol, and BMI*.

**Table 3:** Association of HEI-2015 components with periodontitis.

**HEI-2015 components**	**Unadjusted model**	**Model 1**	**Model 2**	**Model 3**
**Adequacy**	
Total vegetables	0.93 (0.89–0.97); 0.001	0.91 (0.86–0.95); <0.001	0.94 (0.9–0.99); 0.016	0.93 (0.89–0.97); 0.031
Greens and beans	0.95 (0.92–0.98); 0.001	0.94 (0.91–0.97); 0.001	0.98 (0.95–1.02); 0.342	0.94 (0.91–1.03); 0.414
Total fruit	0.96 (0.93–0.99); 0.02	0.93 (0.9–0.96); <0.001	0.92 (0.88–0.95); <0.001	0.96 (0.91–0.99); 0.031
Whole fruits	0.96 (0.93–0.99); 0.013	0.94 (0.91–0.97); <0.001	0.93 (0.9–0.97); <0.001	0.98 (0.92–1.02); 0.054
Whole grains	0.98 (0.96–1); 0.027	0.96 (0.94–0.98); <0.001	0.97 (0.95–1); 0.021	0.96 (0.94–0.98);0.02
Dairy	0.98 (0.96–1);0.033	0.98 (0.96–1); 0.102	1 (0.97–1.02); 0.821	1 (0.98–1.03); 0.733
Total protein foods	0.97 (0.92–1.03); 0.359	0.95 (0.89–1); 0.067	0.93 (0.87–0.99); 0.018	0.97 (0.91–1.21); 0.144
Seafood and plant proteins	0.93 (0.9–0.96); <0.001	0.92 (0.89–0.95); <0.001	0.93 (0.9–0.97); <0.001	0.93 (0.90–0.96); 0.001
Fatty acids	0.97 (0.96–0.99); 0.01	0.97 (0.95–0.99); 0.006	0.96 (0.94–0.98); <0.001	0.96 (0.94–0.98); 0.005
**Moderation**	
Sodium	1 (0.98–1.02);0.782	1.01 (0.98–1.03); 0.614	1 (0.98–1.02); 0.852	0.97 (0.95–1.02); 0.536
Refined grains	0.99 (0.97–1.01); 0.265	0.98 (0.96–1); 0.022	0.99 (0.97–1.02); 0.635	0.97 (0.95–1.02); 0.123
Saturated fats	1.01 (0.99–1.03); 0.328	1.01 (0.99–1.04); 0.234	1.01 (0.99–1.03); 0.497	1.21 (0.99–1.33); 0.213
Added sugars	0.98 (0.96–1.01); 0.137	0.96 (0.94–0.98); <0.001	0.98 (0.95–1); 0.043	0.99 (0.95–1); 0.122

*Model 1: adjusted for gender, age, race*.

*Model 2: Model 1+ adjusted for education, PIR*.

*Model 3: Model 2+smoke, DM, alcohol, and BMI*.

**Table 4:** The actual effect of smoking, age and gender.

**Variable**	**adj. OR_95CI**	**adj. *P*_value**
**Smoke**		
Former	1(Ref)	
Now	2.53 (1.98–3.24)	<0.001
Never	0.73 (0.6–0.88)	0.001
**Age**		
≤ 60	1( Ref)	
>60	1.26 (0.94–1.68)	0.121
**Sex**		
Female	1 (Ref)	
male	1.16 (0.92–1.31)	0.311

### Spline smoothing

**Figure 2:**
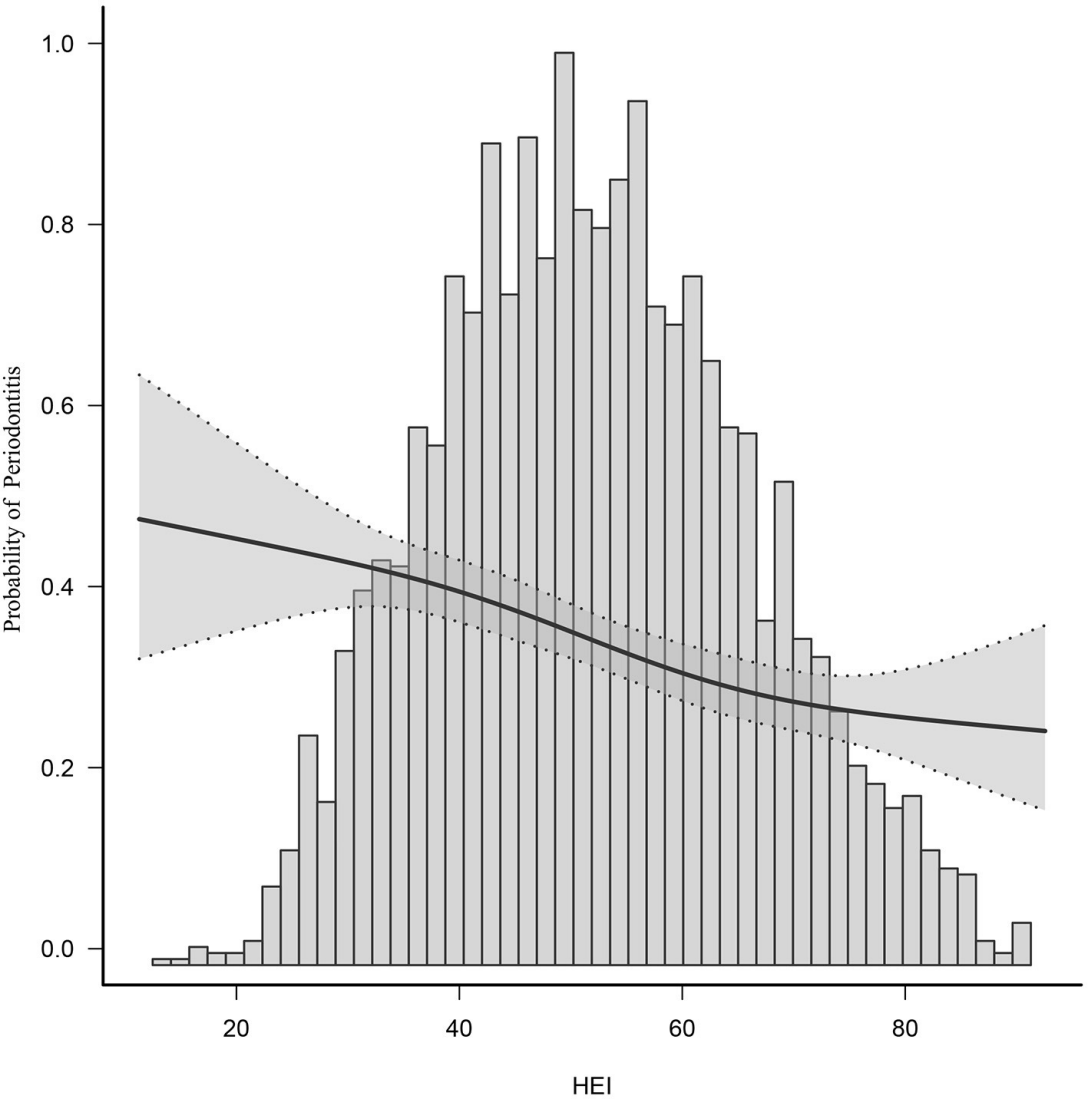
Smooth curve fitting of periodontitis and HEI-2015.

After adjusting for potential confounders, smooth curve fitting indicated a non-linear relationship between 2015 HEI and periodontitis. It is evident from this curve that there is linear relationship between the 2015 HEI and the prevalence of periodontitis (Figure 2). HEI is negatively correlated with periodontitis, i.e., higher HEI is associated with less periodontitis. The middle of the line has a greater slope than the ends of the line.

### Subgroup analyses

To identify potential effect modifiers, we also performed a subgroup analysis (Supplementary Table 4). The results showed that the effect sizes differed significantly across drinking status and DM. For mild drinkers, HEI above 50 (HEI_Q3, HEI_Q4) is protective against periodontitis. Similarly, for participants without diabetes, higher HEI helped to reduce the prevalence of periodontitis. In contrast, among participants with diabetes, the difference was not statistically significant. It is suggested that the promotional effect of diabetes on periodontitis is greater than the protective effect of HEI.

## Discussion

In the cohort we analyzed, 42.9% of the participants had periodontitis, with 8.4% of them having severe periodontitis. Our analysis showed that a higher HEI score was significantly associated with a lower prevalence of periodontitis. People with a better diet quality intake had a lower risk of periodontitis. Of the 13 components of the HEI, fatty acids, seafood and plant proteins, whole grains; total fruit and total vegetables were most associated with periodontitis.

There is an association between periodontitis and diet (20). Previous studies have found that eating naturally fibrous foods reduces plaque build-up and that eating soft foods over a long period of time increases plaque build-up, leading to periodontal disease. In addition, the consumption of a highly inflammatory diet by patients with periodontitis can not only exacerbate clinical symptoms but also increase the risk of other associated systemic diseases (20).

The HEI is a valid and rapid method of assessing the quality of the diet and is made up of the following components: adequacy components (total fruits, whole fruits, total vegetables, greens and beans, whole grains, dairy, total protein foods, seafood and plant proteins, fatty acids) and moderation components (sodium, refined grains, added sugars, saturated fats) (18). Our findings suggest that fatty acids, seafood and plant proteins, whole grains; total fruit and total vegetables are all associated with a lower prevalence of periodontitis. Consumption of potassium-rich foods, such as green vegetables and fruits, has been reported to be effective in reducing the incidence of periodontitis (21). Free fatty acids play a role in the development of periodontitis and omega-3 fatty acids appear to have a positive effect on periodontal wound healing and may reduce periodontitis (22, 23). There are fewer studies on the link between seafood, plant proteins and periodontitis, but previous studies have concluded that a daily intake of ~1 gram of calcium is beneficial for periodontal health and that there is a strong relationship between calcium intake and the development of periodontitis, with lower dietary calcium intake contributing to the incidence of periodontitis (24). Thus, this indirect evidence also suggests that better diet quality is associated with a lower prevalence of periodontitis, which is consistent with our results.

Previous clinical studies have shown that smokers have a higher prevalence of chronic periodontitis, more severe alveolar bone resorption and deeper periodontal pockets, and that smoking is an important contributing factor to chronic periodontitis (25). The analysis showed that the prevalence of periodontitis was higher in participants who were current and former smokers. Non-smoking had a protective effect on periodontitis. The mechanism by which smoking contributes to the development of periodontitis is not yet clear. It has been suggested that smoking inhibits the antimicrobial action of the periodontal gingival sulcus fluid. Smoking can cause changes in inflammatory factors in the gingival sulcus fluid, resulting in an inflammatory response and structural damage to periodontal tissues (26, 27). The negative association between HEI and the risk of periodontitis was more pronounced in those who did not have DM and in those who consumed alcohol lightly. The results suggest that for participants with light alcohol consumption and no DM, obtaining a higher HEI by adjusting their diet may reduce the prevalence of periodontitis.

Previous studies have analyzed the relationship between single nutrients and periodontitis, but the present study is a large population-based cross-sectional study exploring HEI and periodontitis. HEI is a more comprehensive method of nutritional assessment and is more representative of an individual's comprehensive nutritional intake. Therefore, analyzing the relationship between HEI and periodontitis can provide a more comprehensive picture of the relationship between dietary intake and periodontitis. Furthermore, we conclude that the prevalence of periodontitis can be reduced by adjusting dietary intake. Based on a multiple regression analysis of the 13 components of the HEI, we were able to further investigate the relationship between different types of diet and periodontitis.

However, there remain several limitations. Because cross-sectional observational studies cannot indicate causation and directionality, our findings should be interpreted with care. Although confounding has been extensively adjusted for, residual confounding still cannot be fully ruled out. Because of the retrospective nature of the questionnaire, there was a possibility of recall bias on the part of patients.

## Conclusion

Our study found that a lower HEI was associated with a higher incidence of periodontitis, suggesting that a comprehensive health promotion including dietary modification is needed to reduce the burden of periodontal disease in society. Because the findings from this study were cross-sectional, prospective studies are needed to clarify the causal relationship between HEI and periodontitis.

## Data availability statement

The original contributions presented in the study are included in the article/Supplementary material, further inquiries can be directed to the corresponding author.

## Author contributions

All authors made substantial contributions to conception and design, acquisition of data, or analysis and interpretation of data, took part in drafting the article or revising it critically for important intellectual content, agreed to submit to the current journal, gave final approval of the version to be published, and agree to be accountable for all aspects of the work.

## Conflict of interest

The authors declare that the research was conducted in the absence of any commercial or financial relationships that could be construed as a potential conflict of interest.

## Publisher's note

All claims expressed in this article are solely those of the authors and do not necessarily represent those of their affiliated organizations, or those of the publisher, the editors and the reviewers. Any product that may be evaluated in this article, or claim that may be made by its manufacturer, is not guaranteed or endorsed by the publisher.
