# Supplementary material for: The association of healthy eating index with periodontitis in NHANES 2013–2014
Source: Front Nutr. 2022 Aug 9;9:968073. doi: 10.3389/fnut.2022.968073 (PMC9395606; doi:10.3389/fnut.2022.968073)
Supplement: Supplementary file 1 [file Data_Sheet_1.zip › Supplementary Tables/Table S1.docx]

| Classification | Standard |
| --- | --- |
| Mild periodontitis | ≥2 interproximal sites with AL ≥3 mm, and ≥2 interproximal sites with PD ≥4 mm (not on same tooth) or one site with PD ≥5 mm |
| Moderate periodontitis | ≥2 interproximal sites with AL ≥4 mm (not on same tooth), or ≥2 interproximal sites with PD ≥5 mm (not on same tooth) |
| Severe periodontitis | ≥2 interproximal sites with AL ≥6 mm (not on same tooth) and ≥1 interproximal site with PD ≥5 mm |
| No periodontitis | No evidence of mild, moderate, or severe periodontitis |
