# Supplementary material for: The association of healthy eating index with periodontitis in NHANES 2013–2014
Source: Front Nutr. 2022 Aug 9;9:968073. doi: 10.3389/fnut.2022.968073 (PMC9395606; doi:10.3389/fnut.2022.968073)
Supplement: Supplementary file 1 [file Data_Sheet_1.zip › Supplementary Tables/Table S2.docx]

| HEI-2015 Components | Range of Points | Minimum Scoring Standard | Maximum Scoring Standard |
| --- | --- | --- | --- |
| **Adequacy Components** (higher score indicates higher consumption) | | | |
| Total Fruits | 0-5 | 0 | 0.8 cup equiv. /1000 kcal |
| Whole Fruits | 0-5 | 0 | 0.4 cup equiv./1000 kcal |
| Total Vegetables | 0-5 | 0 | 1.1 cup equiv. /1000 kcal |
| Greens and Beans | 0-5 | 0 | 0.2 cup equiv. /1000 kcal |
| Total Protein Foods | 0-5 | 0 | 2.5 oz equiv./1000 kcal |
| Seafood and Plant Proteins | 0-5 | 0 | 0.8 oz equiv./1000 kcal |
| Dairy | 0-10 | 0 | 1.3 cup equiv./1000 kcal |
| Whole Grains | 0-10 | 0 | 1.5 oz equiv. /1000 kcal |
| Fatty Acids ^b^ | 0-10 | (PUFAs + MUFAs)/SFAs ≤1.2 | (PUFAs + MUFAs)/SFAs ≥2.5 |
| **Moderation Components** (higher score indicates lower consumption) | | | |
| Refined Grains | 0-10 | 4.3 oz equiv./1000 kcal | 1.8 oz equiv. /1000 kcal |
| Sodium | 0-10 | 2.0 grams /1000 kcal | 1.1 grams/1000 kcal |
| Added Sugars | 0-10 | 26% of energy | 6.5% of energy |
| Saturated Fats | 0-10 | 16% of energy | 8% of energy |

^a^ Intakes between the minimum and maximum standards are scored proportionately.

^b^ Ratios of polyunsaturated and monounsaturated fatty acids (PUFAs and MUFAs) to saturated fatty acids (SFAs).
