# Supplementary material for: The association of healthy eating index with periodontitis in NHANES 2013–2014
Source: Front Nutr. 2022 Aug 9;9:968073. doi: 10.3389/fnut.2022.968073 (PMC9395606; doi:10.3389/fnut.2022.968073)
Supplement: Supplementary file 1 [file Data_Sheet_1.zip › Supplementary Tables/Table S3.docx]

| \| Variables \| Mean ± SD \| Q1 (n = 750) \| Q2 (n = 750) \| Q3 (n = 750) \| Q4 (n = 751) \| *P* \| \| --- \| --- \| --- \| --- \| --- \| --- \| --- \| \| Total Vegetables \| 3.1 ± 1.7 \| 2.3 ± 1.6 \| 3.0 ± 1.6 \| 3.3 ± 1.6 \| 3.8 ± 1.5 \| < 0.001 \| \| Greens and Beans \| 1.7 ± 2.2 \| 0.6 ± 1.4 \| 1.2 ± 2.0 \| 2.0 ± 2.3 \| 3.1 ± 2.3 \| < 0.001 \| \| Total Fruits \| 2.1 ± 2.1 \| 0.7 ± 1.4 \| 1.7 ± 1.9 \| 2.5 ± 2.0 \| 3.6 ± 1.8 \| < 0.001 \| \| Whole Fruits \| 2.2 ± 2.3 \| 0.7 ± 1.5 \| 1.6 ± 2.1 \| 2.6 ± 2.3 \| 3.8 ± 1.9 \| < 0.001 \| \| Whole Grains \| 2.7 ± 3.5 \| 0.9 ± 1.8 \| 1.8 ± 2.8 \| 3.0 ± 3.6 \| 5.2 ± 3.9 \| < 0.001 \| \| Dairy \| 4.9 ± 3.4 \| 5.1 ± 3.4 \| 4.6 ± 3.4 \| 4.7 ± 3.4 \| 5.1 ± 3.4 \| 0.003 \| \| Total Protein Foods \| 4.3 ± 1.3 \| 3.9 ± 1.5 \| 4.2 ± 1.3 \| 4.4 ± 1.1 \| 4.6 ± 0.9 \| < 0.001 \| \| Seafood and Plant Proteins \| 2.5 ± 2.3 \| 1.0 ± 1.7 \| 2.0 ± 2.2 \| 3.1 ± 2.2 \| 4.0 ± 1.8 \| < 0.001 \| \| Fatty Acids \| 5.3 ± 3.7 \| 2.7 ± 2.8 \| 4.9 ± 3.4 \| 6.1 ± 3.4 \| 7.6 ± 3.1 \| < 0.001 \| \| Sodium \| 4.4 ± 3.5 \| 3.7 ± 3.3 \| 4.2 ± 3.5 \| 4.5 ± 3.4 \| 5.2 ± 3.6 \| < 0.001 \| \| Refined Grains \| 6.1 ± 3.7 \| 4.0 ± 3.6 \| 5.8 ± 3.7 \| 6.6 ± 3.5 \| 8.1 ± 2.7 \| < 0.001 \| \| Saturated Fats \| 6.2 ± 3.5 \| 3.9 ± 3.4 \| 5.9 ± 3.4 \| 6.8 ± 3.2 \| 8.4 ± 2.3 \| < 0.001 \| \| Added Sugars \| 6.9 ± 3.3 \| 5.4 ± 3.6 \| 6.3 ± 3.5 \| 7.4 ± 2.9 \| 8.6 ± 2.1 \| < 0.001 \| |
| --- | --- | --- | --- | --- | --- | --- | --- | --- | --- | --- | --- | --- | --- | --- | --- | --- | --- | --- | --- | --- | --- | --- | --- | --- | --- | --- | --- | --- | --- | --- | --- | --- | --- | --- | --- | --- | --- | --- | --- | --- | --- | --- | --- | --- | --- | --- | --- | --- | --- | --- | --- | --- | --- | --- | --- | --- | --- | --- | --- | --- | --- | --- | --- | --- | --- | --- | --- | --- | --- | --- | --- | --- | --- | --- | --- | --- | --- | --- | --- | --- | --- | --- | --- | --- | --- | --- | --- | --- | --- | --- | --- | --- | --- | --- | --- | --- | --- | --- |
