# Supplementary material for: The association of healthy eating index with periodontitis in NHANES 2013–2014
Source: Front Nutr. 2022 Aug 9;9:968073. doi: 10.3389/fnut.2022.968073 (PMC9395606; doi:10.3389/fnut.2022.968073)
Supplement: Supplementary file 1 [file Data_Sheet_1.zip › Supplementary Tables/Table S4.docx]

| **Subgroup** | **Variable** | **OR_95CI** | ***P*_value** | ***P*.for.interaction** |
| --- | --- | --- | --- | --- |
| **Age** |  |  |  |  |
| ≤60 |  |  |  | 0.99 |
|  | HEI2015_Q1 | 1(Ref) |  |  |
|  | HEI2015_Q2 | 1.01 (0.77~1.31) | 0.958 |  |
|  | HEI2015_Q3 | 0.93 (0.71~1.22) | 0.583 |  |
|  | HEI2015_Q4 | 0.7 (0.53~0.94) | 0.016 |  |
| ＞60 |  |  |  |  |
|  | HEI2015_Q1 | 1(Ref) |  |  |
|  | HEI2015_Q2 | 0.95 (0.63~1.44) | 0.809 |  |
|  | HEI2015_Q3 | 0.91 (0.6~1.36) | 0.635 |  |
|  | HEI2015_Q4 | 0.7 (0.47~1.05) | 0.084 |  |
| **Sex** |  |  |  |  |
| Female |  |  |  | 0.347 |
|  | HEI2015_Q1 | 1(Ref) |  |  |
|  | HEI2015_Q2 | 1.08 (0.79~1.48) | 0.616 |  |
|  | HEI2015_Q3 | 0.84 (0.61~1.16) | 0.291 |  |
|  | HEI2015_Q4 | 0.67 (0.48~0.93) | 0.018 |  |
| Male |  |  |  |  |
|  | HEI2015_Q1 | 1(Ref) |  |  |
|  | HEI2015_Q2 | 0.91 (0.66~1.24) | 0.542 |  |
|  | HEI2015_Q3 | 1.04 (0.76~1.42) | 0.796 |  |
|  | HEI2015_Q4 | 0.77 (0.56~1.07) | 0.118 |  |
| **Smoke** |  |  |  |  |
| former |  |  |  | 0.111 |
|  | HEI2015_Q1 | 1(Ref) |  |  |
|  | HEI2015_Q2 | 0.84 (0.54~1.3) | 0.434 |  |
|  | HEI2015_Q3 | 1.09 (0.71~1.69) | 0.681 |  |
|  | HEI2015_Q4 | 0.98 (0.63~1.54) | 0.934 |  |
| never |  |  |  |  |
|  | HEI2015_Q1 | 1(Ref) |  |  |
|  | HEI2015_Q2 | 1.08 (0.79~1.47) | 0.641 |  |
|  | HEI2015_Q3 | 0.93 (0.68~1.27) | 0.641 |  |
|  | HEI2015_Q4 | 0.7 (0.51~0.95) | 0.023 |  |
| now |  |  |  |  |
|  | HEI2015_Q1 | 1(Ref) |  |  |
|  | HEI2015_Q2 | 1.07 (0.67~1.7) | 0.79 |  |
|  | HEI2015_Q3 | 0.75 (0.44~1.28) | 0.292 |  |
|  | HEI2015_Q4 | 0.49 (0.26~0.91) | 0.023 |  |
| **Alcohol.user** |  |  |  |  |
| former |  |  |  | **0.042** |
|  | HEI2015_Q1 | 1(Ref) |  |  |
|  | HEI2015_Q2 | 0.85 (0.5~1.47) | 0.57 |  |
|  | HEI2015_Q3 | 1.38 (0.81~2.36) | 0.234 |  |
|  | HEI2015_Q4 | 1.16 (0.67~2) | 0.594 |  |
| heavy |  |  |  |  |
|  | HEI2015_Q1 | 1(Ref) |  |  |
|  | HEI2015_Q2 | 1.49 (0.92~2.4) | 0.103 |  |
|  | HEI2015_Q3 | 1.39 (0.84~2.3) | 0.203 |  |
|  | HEI2015_Q4 | 0.61 (0.34~1.11) | 0.107 |  |
| mild |  |  |  |  |
|  | HEI2015_Q1 | 1(Ref) |  |  |
|  | HEI2015_Q2 | 0.75 (0.51~1.11) | 0.152 |  |
|  | HEI2015_Q3 | 0.58 (0.39~0.86) | 0.007 |  |
|  | HEI2015_Q4 | 0.61 (0.42~0.9) | 0.014 |  |
| moderate |  |  |  |  |
|  | HEI2015_Q1 | 1(Ref) |  |  |
|  | HEI2015_Q2 | 0.88 (0.51~1.54) | 0.661 |  |
|  | HEI2015_Q3 | 0.96 (0.52~1.75) | 0.882 |  |
|  | HEI2015_Q4 | 0.58 (0.31~1.09) | 0.092 |  |
| never |  |  |  |  |
|  | HEI2015_Q1 | 1(Ref) |  |  |
|  | HEI2015_Q2 | 1.57 (0.83~2.98) | 0.165 |  |
|  | HEI2015_Q3 | 1.15 (0.62~2.11) | 0.661 |  |
|  | HEI2015_Q4 | 0.68 (0.36~1.3) | 0.249 |  |
| **DM** |  |  |  |  |
| DM |  |  |  |  |
|  | HEI2015_Q1 | 1(Ref) |  | **0.027** |
|  | HEI2015_Q2 | 0.71 (0.4~1.23) | 0.221 |  |
|  | HEI2015_Q3 | 1.28 (0.72~2.29) | 0.395 |  |
|  | HEI2015_Q4 | 1.12 (0.61~2.03) | 0.719 |  |
| no |  |  |  |  |
|  | HEI2015_Q1 | 1(Ref) |  |  |
|  | HEI2015_Q2 | 1.06 (0.84~1.35) | 0.614 |  |
|  | HEI2015_Q3 | 0.88 (0.69~1.12) | 0.29 |  |
|  | HEI2015_Q4 | 0.66 (0.51~0.84) | 0.001 |  |
| **Poverty** |  |  |  |  |
| high |  |  |  | 0.603 |
|  | HEI2015_Q1 | 1(Ref) |  |  |
|  | HEI2015_Q2 | 0.91 (0.57~1.44) | 0.681 |  |
|  | HEI2015_Q3 | 1.06 (0.67~1.66) | 0.816 |  |
|  | HEI2015_Q4 | 0.91 (0.59~1.41) | 0.683 |  |
| low |  |  |  |  |
|  | HEI2015_Q1 | 1(Ref) |  |  |
|  | HEI2015_Q2 | 1.31 (0.9~1.91) | 0.164 |  |
|  | HEI2015_Q3 | 1.05 (0.72~1.55) | 0.788 |  |
|  | HEI2015_Q4 | 0.87 (0.56~1.36) | 0.538 |  |
| medium |  |  |  |  |
|  | HEI2015_Q1 | 1(Ref) |  |  |
|  | HEI2015_Q2 | 1.03 (0.71~1.49) | 0.872 |  |
|  | HEI2015_Q3 | 0.93 (0.64~1.36) | 0.724 |  |
|  | HEI2015_Q4 | 0.74 (0.5~1.09) | 0.123 |  |
